# Looks like Tuberculous Meningitis, But Not: A Case of Rhinocerebral Mucormycosis with Garcin Syndrome

**DOI:** 10.3389/fneur.2016.00181

**Published:** 2016-10-24

**Authors:** HongNa Yang, CuiLan Wang

**Affiliations:** ^1^Department of Critical Care Medicine, Qilu Hospital of Shandong University, Shandong University, Jinan, Shandong Province, China; ^2^Department of Neurology, Qilu Hospital of Shandong University, Shandong University, Jinan, Shandong Province, China; ^3^Brain Science Research Institute, Shandong University, Jinan, Shandong Province, China

**Keywords:** rhinocerebral mucomycosis, tuberculous meningitis, Garcin syndrome

## Abstract

Rhinocerebral mucomycosis (RCM) as an emerging opportunistic, angioinvasive, and devastating fungi infection with high mortality is difficult to be diagnosed early because of the lack of specific clinical features or manifestations. Garcin syndrome is more often caused by skull base and rhinopharyngeal tumors or metastases, and basal meningitis. We reported that an aged diabetic man, involved nearly all cranial nerves (Garcin syndrome), who was at first suspected to be suffered from tuberculous meningitis, ultimately developed typically progressing RCM. Diagnosis was made to find the presence of mucormycosis in the infected tissue by biopsy.

## Introduction

Mucormycosis, ubiquitous in the environment, is known as an emerging opportunistic, angioinvasive, and devastating fungi infection with high mortality. Mucormycosis is divided into six major forms according to the presentation and anatomic site, which include rhinocerebral, pulmonary, cutaneous, gastrointestinal, disseminated, and uncommon rare forms, such as endocarditis, osteomyelitis, peritonitis, and renal infection (1). Malignant hematological disease with or without stem cell transplantation, prolonged and severe neutropenia, poorly controlled diabetes mellitus with or without diabetic ketoacidosis, iron overload, major trauma, prolonged use of corticosteroids, illicit intravenous drug use, neonatal prematurity, and malnourishment are known as the predisposing disorders to mucormycosis (1). More importantly, diabetic mellitus is the most prevalent underlying disease to mucomycosis, especially to rhinocerebral mucomycosis (RCM) (1). In addition, the identification of the fungal agent in infected tissues is now still the only golden standard to diagnose mucormycosis because of the lack of specific clinical features or manifestations, despite the determination of 16s rRNA gene sequence of mucormycosis in CSF (cerebrospinal fluid) by PCR (polymerase chain reaction) was proposed as the early diagnosis of RCM (2). Thus, it is difficult to diagnose the RCM in time.

Garcin syndrome is characterized by (1) progressive involvement of the cranial nerves culminating in total unilateral paralysis of all or nearly all cranial nerves, (2) lack of sensor-motor signs in the extremities, (3) absence of increased intracranial pressures, and (4) X-ray evidence of osteoclastic lesions at the skull base (3). In addition, Garcin syndrome is more often caused by skull base and rhinopharyngeal tumors or metastases, and basal meningitis (4). However, to our limited knowledge, there were little case reports about RCM with Garcin syndrome. Especially, all the cranial nerves were involved (4). This clinical report illustrated that an aged diabetic man, involved nearly all cranial nerves (Garcin syndrome), who was at first highly suspected to be suffered from tuberculous meningitis (TBM), ultimately developed typically progressing RCM.

## Case Presentation

We got the oral consent from the patient’s daughter by cell phone. The patient, with a medical history of type 2 diabetes mellitus, pulmonary tuberculous, and hypertension, was admitted to neurological department of Qilu Hospital of Shandong University (Jinan, China) for pain on the left head accompanying with left facial numbness of 10 days’ duration. He also suffered from central retina vein occlusion 5 years ago. In addition, he also complained associated dysphagia, drinking cough, and ageusia. The left facial numbness became progressively worse 2 days before admission, which was associated with dizziness and unsteady walk. When he was admitted to the hospital, the temperature was 37.6°C. Throughout the period of hospitalization, the temperature of patient did not exceed 38°C. Left ptosis, the loss of left pupillary light reflex, corneal reflex, and pharyngeal reflex were detected. The diameter of left pupil (6 mm) was larger than the right pupil (3 mm). In addition, left eye movement was restricted to all directions. The jaw deviated to the left on mouth opening. The movement of soft palate and pharynx was diminished on the left side. The hearing was not affected by Weber’s test and Rinne’s test. Barnv test and Hallpike maneuver test were not performed to test vestibular function. The tongue was deviated to the left on protrusion. Left Babinskin sign was positive. Stiff neck and meningeal irritation were not elicited. The power and deep tendon reflexes were essentially normal. The rest of the systemic examinations were unremarkable. Above these physical examinations indicated that III, IV, V, VI, VII, IX, X, and XII (oculomotor, trochlear, trigeminal, abducens, facial, glossopharyngeal, vagus, and hypoglossal) nerves were impaired. More importantly, the impaired cranial nerves were unilateral or left.

Lumbar puncture revealed a turbid fluid with a mixed leukocytic pleocytosis of 546 leukocytes/μl (38% neutrophils, 61% lymphocytes, and 1% monocytes), markedly elevated IgG of 953.00 mg/l (normal value <34 mg/l), and moderately elevated IgM of 10.00 mg/l (normal value <1.3 mg/l) as well as IgA of 28.80 mg/l (normal value <5 mg/l). But the level of CSF protein was normal. In addition, the levels of lactate, glucose, and Cl^−^ in the CSF are under the normal CSF levels. But the pressure of CSF was normal. Oligoclonal band in the CSF is negative. The tuberculin skin test was positive. Blood examination showed the elevated white blood cells (WBC) count of 15.75 × 10^9^/l (90.7% neutrophils) and ESR: 37 mm/h. Blood glucose was always higher than 16 mmol/l and lower than 20 mmol/l during the hospital. Hepatic and renal functions were not impaired. Computed tomography (CT) and magnetic resonance imaging (MRI) revealed that the walls of maxillary sinus, ethmoid sinus, sphenoid sinus, and frontal sinus were intact except for the thickening mucosa. In addition, there was no involvement of brain on CT or MRI. He was highly suspected to suffer from TBM. Thus, he was under anti-tuberculous therapy with isoniazid, rifampin, and pyrazinamide, as well as low-dose corticosteroid therapy (10 mg dexcimethasome per day, IV drip) according to the data from CSF and clinical manifestations. However, the clinical manifestations were not remitted after anti-tuberculous therapy treatment.

**Figure 1:**
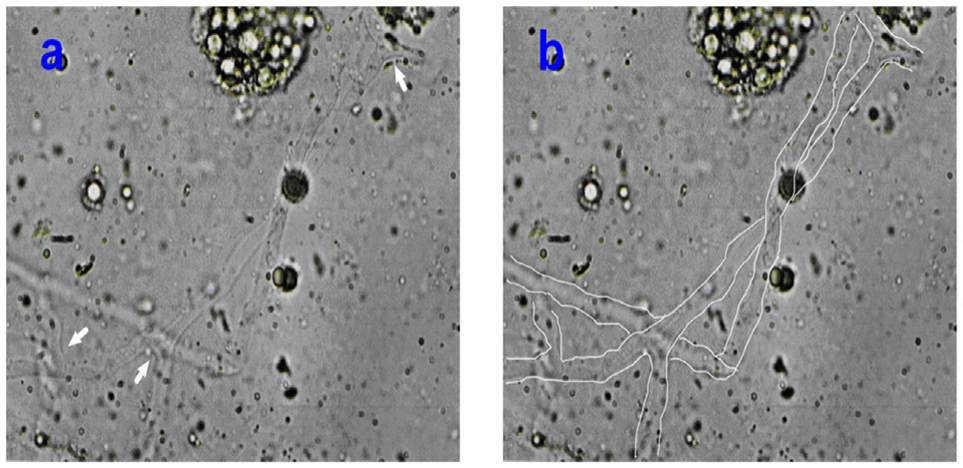
**(A,B)** The pictures illustrated the non-septate wide ribbon-shape mucormycotic hyphae observed in KOH (20%) preparation under 400× magnification. The arrows **(A)** indicated the rectangular divides of hyphae. The mucormycotic hyphae were drawn in white line.

On the sixth day of admission to the hospital, the patient complained the pain in the mouth. Thus, we found that there was about 2 cm × 1.5 cm ulcer in the left hard plate mucosa. So, the biopsy was advised. The left hard plate mucosa with the adjacent soft tissue was excised under local anesthesia. Non-septate wide hyphae was observed under microscope by KOH (20%) preparation of specimen, indicating mucormycosis (Figure 1).

After 3 days of treatment with amphotericin B, the patient refused further surgical treatment as well as combination antifungal therapy, and voluntarily discharged although the patient was informed other possible interventions for the disease and probable outcome after discharge. After 7 days of discharge, the patient died with black necrotic eschar. The patient’s relatives refused to do postmortem examination.

## Discussion

In this case, the patient was at first highly suspected to be suffered from TBM according to clinical manifestation (low fever and cranial nerve paralysis), tuberculin skin test, as well as medical history, although the presentation of CSF was atypic. But unilateral cranial nerve paralysis could not be completely explained by TBM. It is well accepted that basilar meningeal fibrosis and vascular inflammation of TBM resulted in bilateral cranial nerves paralysis (5). We are not sure whether VIII was affected because vestibular functions and audiometry were not tested, which is our limitation. However, nearly all unilateral cranial nerves paralysis (III, IV, V, VI, VII, IX, X, and XII) still indicated the presence of Garcin syndrome. It is well accepted that the most prevalent cause of Garcin syndrome is skull base and rhinopharyngeal tumors or metastases, and basal meningitis (4). In addition, there were no problems except for the thickening mucosa of maxillary sinus, ethmoid sinus, sphenoid sinus, and frontal sinus on the first day of admission to the hospital. To date, there were little case reports about Garcin syndrome in patients with RCM. Thus, at first, we did not consider the presence of Garcin syndrome and treated the patient under anti-tuberculous therapy. However, the clinical manifestations were not remitted after anti-tuberculous therapy treatment. We only realized the presence of RCM until we saw the ulcer in the left hard plate mucosa and found the mucormycosis in the infected tissue by biopsy. Mucormycosis can exhibit angiocentric growth resulting in thrombosis of sinus or internal carotid artery (1), which could not be the explanation for unilateral cranial nerves paralysis. It was estimated that the mycelial growth along nerves or leptomeningeal vessels resulted in the strict unilateral cranial nerves involvement. We described the comparisons between TBM and RCM in Table 1.

**Table 1:** **Clinical features of tuberculous meningitis and rhinocerebral mucomycosis**.

	Tuberculous meningitis	Rhinocerebral mucormycosis
Area	Developing countries most common	Developing countries or developed countries
Predisposing disorders	Elderly, immunocompromised patient	Malignant hematological disease with or without stem cell transplantation, prolonged and severe neutropenia, poorly controlled diabetes mellitus with or without diabetic ketoacidosis, iron overload, major trauma, prolonged use of corticosteroids, illicit intravenous drug use, neonatal prematurity, and malnourishment
Symptoms	Prodromal period with low-grade fever, malaise, weight loss, vomiting, confusion, and coma	Nasal congestion, headache, earache, ophthalmoplegia, unilateral periorbital facial pain, acute vision loss, cerebral infarction or hemorrhage, and sagittal sinus thrombosis
Clinical findings	Cranial nerve palsies (VI, III, and IV), focal neurological signs, stiff neck (common), and urinary retention	Multiple cranial nerve palsies, periorbital edema, proptosis, black necrotic intranasal, or palatal eschar (most common)
Diagnosis	Lumbar puncture preferred	Biopsy analysis of the suspected areas of infections first recommended
		CT for detecting destruction of periorbital tissues and bone
		MRI for identifying the intradural and intracranial extent of ROCM, cavernous sinus thrombosis, and thrombosis of cavernous portions of the internal carotid artery
CSF findings	Raised pressure, raised white cell count (0.05–1 × 10^9^/l) with neutrophils and lymphocytes, raised protein	Atypical
Treatment	2-month initiation phase with four drugs (rifampicin, isoniazid, pyrazinamide, and ethambutol) followed by a 10-month continuation phase of two drugs (rifampicin and isoniazid)	Liposomal amphotericin B (5–10 mg/kg/day) combination with extensive early surgical debridement
Outcome	Mortality <15%, but high disability	Most die

To date, early diagnosis, early medical interventions with amphotericin B and wide-spread surgical debridement of the infected area as well as active reversal of the underlying predisposing factors are still considered to be effective methods to improve the survival rate of mucormycosis, despite FDA recently has approved deferasirox as a promising agent to impact outcome of mucomycosis for clinical use in Europe and India (1, 6). Clinicians, especially neurologists, rarely realize the presence of RCM because the diagnosis of RCM was still relying on the presence of mucomycosis in the infected tissues by the biopsy. Thus, it brought difficulties for neurologists to diagnose RCM. Although we diagnosed RCM early and used anti-fungi therapy with amphotericin B, the patient died because of his worse compliance and continuously aggravating process of the disease.

## Conclusion

Clinicians should be especially aware that rhinocerebral mucormycosis should be considered in the elderly diabetic patient with Garcin syndrome.

## Author Contributions

HY wrote the manuscript. CW collected these clinical data.

## Conflict of Interest Statement

The authors report no conflicts of interest. The authors alone are responsible for the content and writing of the paper.

## References

[1] Petrikkos G Skiada A Lortholary O Roilides E Walsh TJ Kontoyiannis DP. Epidemiology and clinical manifestations of mucormycosis. Clin Infect Dis (2012) 54:S23–34. 10.1093/cid/cir866 22247442

[2] Bengel D Susa M Schreiber H Ludolph AC Tumani H. Early diagnosis of rhinocerebral mucormycosis by cerebrospinal fluid analysis and determination of 16s rRNA gene sequence. Eur J Neurol (2007) 14:1067–70. 10.1111/j.1468-1331.2007.01878.x 17718704

[3] Hakusui S Fujishiro K Takahashi A. An autopsied case of primary epipharyngeal rhabdomyosarcoma presenting Garcin syndrome. Jpn J Med (1991) 30:379–82. 10.2169/internalmedicine1962.30.379 1942655

[4] Hanse MCJ Nijssen PCG Unilateral palsy of all cranial nerves (Garcin syndrome) in a patient with rhinocerebral mucormycosis. J Neurol (2003) 250:506–7. 10.1007/s00415-003-1019-y 12760392

[5] Marx GE Chan ED Tuberculous meningitis: diagnosis and treatment overview. Tuberc Res Treat (2011) 2011:1–9. 10.1155/2011/798764 PMC3335590 22567269

[6] Ibrahim AS Spellberg B Edwards J. Iron acquisition: a novel perspective on mucormycosis pathogenesis and treatment. Curr Opin Infect Dis (2008) 21:620–5. 10.1097/QCO.0b013e3283165fd1 18978530 PMC2773686

